# Can young‐of‐the‐year invasive fish keep up with young‐of‐the‐year native fish? A comparison of feeding rates between invasive sticklebacks and whitefish

**DOI:** 10.1002/ece3.8486

**Published:** 2022-01-23

**Authors:** Žiga Ogorelec, Lars G. Rudstam, Dietmar Straile

**Affiliations:** ^1^ Limnological Institute University of Konstanz Konstanz Germany; ^2^ National Institute of Biology Ljubljana Slovenia; ^3^ Department of Natural Resources and the Environment Cornell Biological Field Station Cornell University Bridgeport New York USA

**Keywords:** competition, fish size, gape limitation, planktivory, resource use, seasonality, stickleback, whitefish, zooplankton

## Abstract

Invasion of non‐native species might alter food web structure and the strength of top‐down control within lake ecosystems. As top‐down control exerted by fish populations is often dominated by young of the year fish, the impact of new fish species might depend on the feeding rates of the juvenile fish. Here we provide comparative analyses of feeding rates of juvenile whitefish (*Coregonus wartmanni*) – a native and specialised planktivore and an invasive generalist (sticklebacks, *Gasterosteus aculeatus*). We studied feedings rates of whitefish and sticklebacks in aquaria experiments using 2 cm to 8 cm fish feeding on seven zooplankton species common to Lake Constance. As whitefish hatch several months earlier than sticklebacks, 0+ whitefish are larger than 0+ sticklebacks throughout the year and hence are predicted to have higher feeding rates on especially large zooplankton species. We show that sticklebacks as small as 2 cm were able to feed on the largest zooplankton species of Lake Constance. Further, stickleback feeding rates were similar to both the same size 0+ whitefish and the larger 0+ whitefish co‐occurring with smaller 0+ sticklebacks. Hence, 0+ sticklebacks will compete with 0+ whitefish for the same zooplankton species, therefore the invasion of sticklebacks is unlikely to change the relative feeding pressure by individual 0+ fish on zooplankton species.

## Graphical abstract

The juvenile period of fish is crucial for their establishment in a new habitat as well as for the grazing impact of fish on prey communities. Here, we show that juveniles of an invasive species, sticklebacks, do have similar feeding rates on various zooplankton species compared to native whitefish despite the latter are specialized planktivores and advanced sticklebacks in growth during the seasonal course. Our results suggest that the invasion of sticklebacks and partial replacement of whitefish should not result in changing relative feeding pressures on the various zooplankton species.

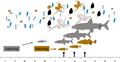

## 1 INTRODUCTION

The age structure and demography of invasive species can be important factors determining invasion success (Ernandes‐Silva et al., 2016; Järemo & Bengtsson, 2011). Likewise, food web effects of invasive species might depend on the age structure of the invasive population and possible differences in food‐web interaction between juveniles and adults of the invasive species. Knowledge of juvenile feeding rates might be especially important for invasive planktivorous fish, as juvenile fish often dominate predation pressure on zooplankton (Mehner & Thiel, 1999; Sommer et al., 2012).

Fish predation is an important structuring force of zooplankton communities, influencing zooplankton size structure and species composition (Brooks & Dodson, 1965). Fish predation also affects seasonal succession (Gliwicz & Pijanowska, 1989) and depth distribution (Gliwicz, 1986) of zooplankton and ‐ via cascading interactions –of phytoplankton in lakes (Hansson et al., 2004; Ogorelec et al., 2021). Consequently, changes in fish predation pressure due to the invasion of a new fish species may have pronounced effects on zooplankton assemblages (Bøhn & Amundsen, 1998; Florian et al., 2016; Nobre et al., 2019).

Ontogenetic growth of 0+ fish (young‐of‐the‐year) is associated with a rapid change in zooplankton species selection (Hartmann, 1983; Makrakis et al., 2008), and seasonal changes in predation pressures on individual zooplankton species (Mehner & Thiel, 1999). During ontogenetic growth, 0+ fish increase their gape size and switch from small prey items such as ciliates and rotifers to increasingly larger crustacean zooplankton species (Gliwicz & Pijanowska, 1989; Zingel et al., 2012). Assuming similar growth rates, the timing when 0+ fish are able to consume zooplankton of a specific size will also depend on the hatching phenology of the fish species and – all other things equal ‐ early hatching fish are predicted to feed earlier in the season on large zooplankton compared to late hatching fish. Hence, predation impact on specific zooplankton species by 0+ fish will change during the season and depend strongly on the growth rates and life histories of the fish species. However, after overcoming gape limitation, 0+ fish may dominate predation pressure on zooplankton relative to their older conspecifics (Mehner & Thiel, 1999; Sommer et al., 2012). Hence, knowledge on the feeding rates of 0+ fish is necessary for assessing the potential impact of invasive fish species in their new habitat.

Historically the pelagic fish community and fish predation pressure on zooplankton in Lake Constance (Germany, Switzerland and Austria) was dominated by whitefish ‐ the Blaufelchen (*Coregonus wartmanni*; Eckmann & Rösch, 1998; Eckmann et al., 2002). However, starting in 2012/2013 the pelagic zone was invaded by sticklebacks (*Gasterosteus aculeatus*), which numerically dominated the fish community in recent years (Eckmann & Engesser, 2019; Hudson et al., 2021; Rösch et al., 2018). The two fish species differ strongly in morphological and behavioural specialisation to the pelagic habitat. Whitefish are characterised by rounded terminal mouths, 29–46 gill rankers, and the swim‐search method (Kottelat & Freyhof, 2007; Lazzaro, 1987). This makes them specialised planktivores and therefore they should be more efficient in zooplankton consumption in the pelagic zone than non‐specialised fish (Lazzaro, 1987; Svärdson, 1976). In contrast, sticklebacks are feeding generalists with only 17–25 gill rankers, occupy diverse habitats and consume a wide range of prey (Kottelat & Freyhof, 2007; Morrow, 1980).

**FIGURE 1:**
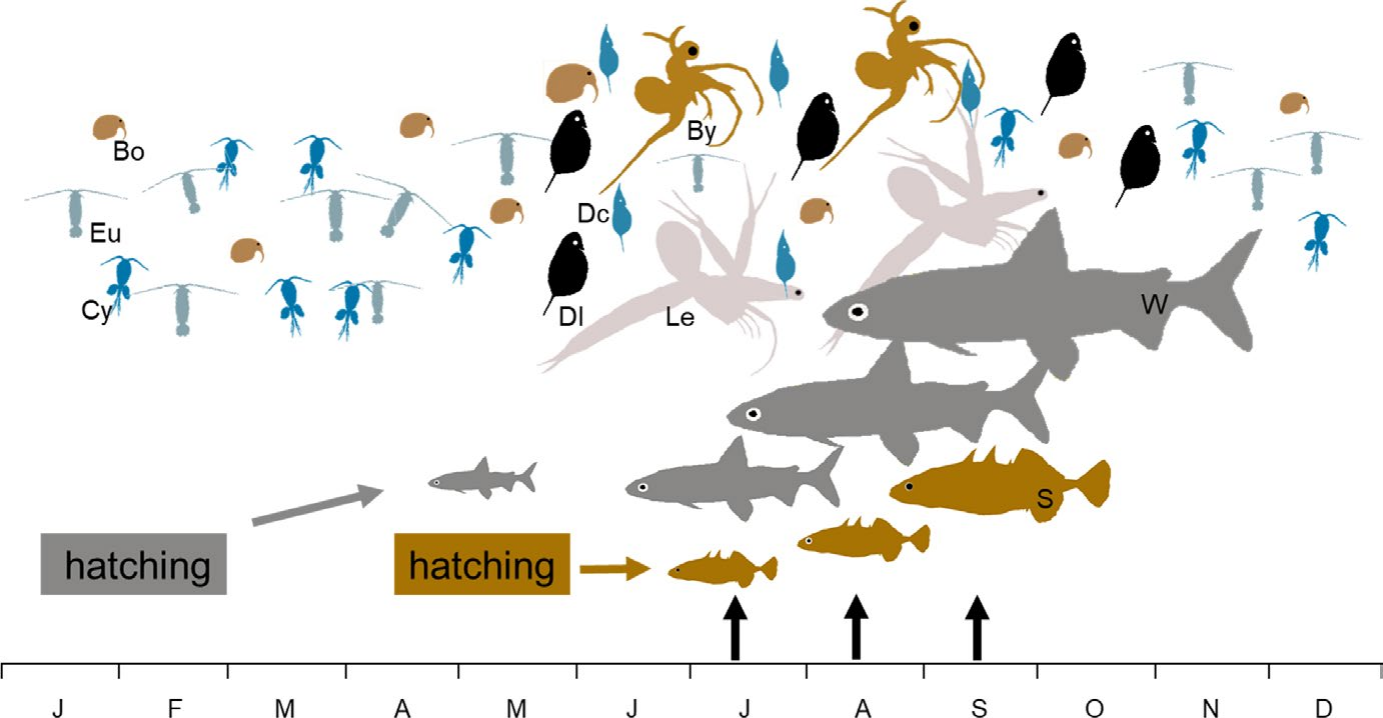
Seasonal size changes of whitefish (W) and stickleback (S) young‐of‐the‐year and zooplankton (*Bosmina* (Bo), *Eudiaptomus* (Eu), cyclopoids (Cy), *Daphnia longispina* (Dl), *Daphnia cucullata* (Dc), *Leptodora* (Le), *Bythotrephes* (By)) seasonality in Lake Constance. Black arrows indicate fish size pairs which feeding rates were compared using ANOVA. Zooplankton sizes are shown enlarged by approximately a factor of 10 compared to fish sizes

The phenology of the two species also differs (Figure 1). Whitefish spawn in December of the preceding year and hatch in February (Eckmann & Rösch, 1998; Kopfmüller & Scheffelt, 1924; Straile et al., 2015), whereas sticklebacks start to hatch in late May (Gugele et al., 2020; Kottelat & Freyhof, 2007) after a short egg development period of approximately one week. Consequently, the size of 0+ whitefish exceeds the size of 0+ sticklebacks throughout the season. Furthermore, adult sticklebacks but not whitefish perform a spawning migration to the lakeshore in spring (Gugele et al., 2020), which further shifts the age composition of pelagic sticklebacks during summer towards dominance of 0+ fish (Gugele et al., 2020). Predator size will affect the species of zooplankton that are consumed, therefore, we expect a lake with planktivory dominated by 0+ whitefish would have different seasonal changes in predation pressure on individual zooplankton species than a lake dominated by 0+ sticklebacks.

Here we study feeding rates of 2 to 8 cm 0+ whitefish and sticklebacks on the seven most abundant crustacean zooplankton species in Lake Constance (Figure 1). We used aquaria experiments to test the hypothesis that: (1) at equal size, the specialised planktivorous whitefish will have higher feeding rates on zooplankton species compared to the generalist sticklebacks; (2) the feeding rate differences between individuals of the two species co‐occurring in time (on average 0+ whitefish size always exceeds 0+ stickleback size in a specific month) will be larger than those between similar‐sized individuals; (3) co‐occurring fish differ in feeding rates on zooplankton species.

## 2 METHODS

### 2.1 Fish used in experiments

Offspring from wild‐caught Lake Constance whitefish were obtained from the Fish Breeding Station Baden‐Württemberg in Langenargen after their hatching at the end of March 2018. They were transported to the Limnological Institute, University of Konstanz, where experiments were performed. Young whitefish were raised until September, at which time some of them reached 8 cm total length. Sticklebacks (3–7 cm) were caught from Lake Constance in spring and summer 2018, while smaller sticklebacks were obtained by inducing spawning via an increase of water temperatures in aquaria. Both fish species were reared in multiple 21 L and 100 L plastic round tanks and fed brine shrimp (*Artemia salina*) during the first two weeks of life followed by live or frozen zooplankton from the lake. The initial number was more than 300 individuals of each species.

### 2.2 Experimental setup

Experiments were conducted in plastic aquariums with dimensions of 20.5 × 38.5 × 25.0 cm, filled with 16 L lake water, filtered through a sieve of 100 μm mesh size. All sides were painted with black tinting colour to reduce disturbances, except for the front side to enable observations. The experimental temperature was kept constant at 15.5°C ± 1°C across the experiments to not confound the effect of fish size in statistical analyses. Light intensity in the middle of aquaria was 250–300 lux. The light was regulated by Sera Digital Dimmer, and the duration of a light cycle was adapted to natural conditions, lasting 13–17 h per day, with a 30–60 min transition period, depending on the season. Aquaria were illuminated by halogen lamps Sera cool daylight 1120, with a colour temperature 10,000 to 12,000 Kelvin. This light simulated the blue spectrum of natural pelagic habitat since whitefish tend to feed in depths between 15 m the night and 35 m during daytime according to average year‐round population depths (Helland et al., 2007; Ohlberger et al., 2008). In upper Lake Constance, both sticklebacks and whitefish are most abundant in depths from 12 to 35 m (Alexander et al., 2016), or from 9 to 18 m for stickleback (Gugele et al., 2020). Individual fish were introduced and left in experimental aquariums overnight before their trial to enable fish adaptation to the new environment and standardize appetite. Each experimental trial included only one zooplankton species and one fish in order to get prey species‐specific feeding rates for each fish group. We used four size classes of both fish species (2, 3, 4 and 6 cm (± 0.25 cm) total length) and an additional 8 cm (± 0.25 cm) in the case of whitefish. Experiments were performed from May to September 2018 in strict accordance with the Protection of Animals Act Germany. The protection was approved by the regional council of Freiburg (reference number 35‐9185.81/G‐17/119).

Seven of the most abundant zooplankton taxa of Lake Constance were used as prey: *Bosmina* spp., *Daphnia cucullata*, and *Daphnia longispina* were reared in the laboratory, while *Eudiaptomus gracilis*, cyclopoid copepods, *Leptodora kindtii*, and *Bythotrephes longimanus* were caught from the lake and then separated and counted in the laboratory. The smallest whitefish size category (2 cm) was not given *L*. *kindtii* because this prey type was too rare in the lake when the 2 cm whitefish were available. Each treatment had six replicates. The only two exceptions were trials with 3 cm whitefish feeding *D*. *longispina* and 4 cm whitefish feeding *E*. *gracilis* with only 5 trials. In total this resulted in 370 feeding trials (9 fish species – size class combinations × 7 zooplankton species × 6 replicates – 2 missing replicates – 6 × 2 cm whitefish feeding *L*. *kindtii*). Each trial was conducted with 32 individuals of each zooplankton species (2 ind/L). The prey was poured through a tube into the middle of the aquaria, which enabled a quick dispersion of zooplankton. Feeding events were observed and counted by one person sitting in front (1 m distance) of the aquaria. Recording started after the fish made a successful bite and continued for 3 min, during which the number of successful bites was recorded. In cases when there was no prey eaten within 5 min after introducing the zooplankton, the number of bites was marked as 0. After the experiment, fish were returned to the main holding tank that contained 300+ individual fish. Consequently, larger (older) fish might have been used already at a smaller size. However, as fish used in trials had weeks to re‐acclimate to the tanks before a possible re‐use, we considered all feeding trials as independent observations in statistical analyses.

102 fish were euthanized by use of 2 g/L TCMP (1,1,1–Trichloro‐2‐methyl‐2‐propanol hemihydrate) and preserved in 70% EtOH to measure the widest dimensions (to the nearest 0.1 mm) of the extended gape with a calliper. Zooplankton body size was measured at three sampling occasions, from a random sample of zooplankton prepared for experiments, and at least ten individuals per species.

### 2.3 Data analysis

Prey‐specific feeding rates were calculated as number of consumed prey per three minutes after the first bite for statistical analysis and divided by three for graphical display to obtain feeding rates per minute. We used zero‐inflated negative binomial (ZINB) models to analyse prey‐specific feeding rate differences between whitefish and sticklebacks. ZINB models were used to account for a high amount of zeros and overdispersion. ZINB models are mixture models combining a negative binomial count distribution with a logistic model to account for excess zeros (Zeileis et al., 2008). We compared the performance of models with different combinations of predator ID and prey ID in the negative binomial and logistic part of the model based on Akaike's information criterion (AIC). Subsequently, we used likelihood ratio tests to test for the significance of individual predictors by comparing the best models based on AIC with reduced models lacking the predictor of interest. ZINB models were in all cases superior to normal negative binomial models. Bootstrap confidence intervals for the predictions of the NB and logit part of the ZINB models were calculated using the R boot package (Canty & Ripley, 2020). We considered differences in feeding rates on different zooplankton species significant when the 95% confidence intervals did not overlap.

The same tests were used to evaluate differences in feeding rates between the size groups of whitefish and sticklebacks which overlap seasonally in the lake. Based on the seasonal increase in lengths in the lake (Eckmann et al., 2007; Gugele et al., 2020), we compared 2 cm sticklebacks with 4 cm whitefish (representing July, Figure 1), 3 cm sticklebacks with 6 cm whitefish (representing August) and 4 cm sticklebacks with 8 cm whitefish (representing September). All analyses were done in R (R Core Team, 2018), using packages *lmtest* (Zeileis & Hothorn, 2002), *boot* (Canty & Ripley, 2020), *pscl* (Zeileis et al., 2008), *plotrix* (Lemon, 2006) and *scales* (Wickham & Seidel, 2020).

## 3 RESULTS

**FIGURE 2:**
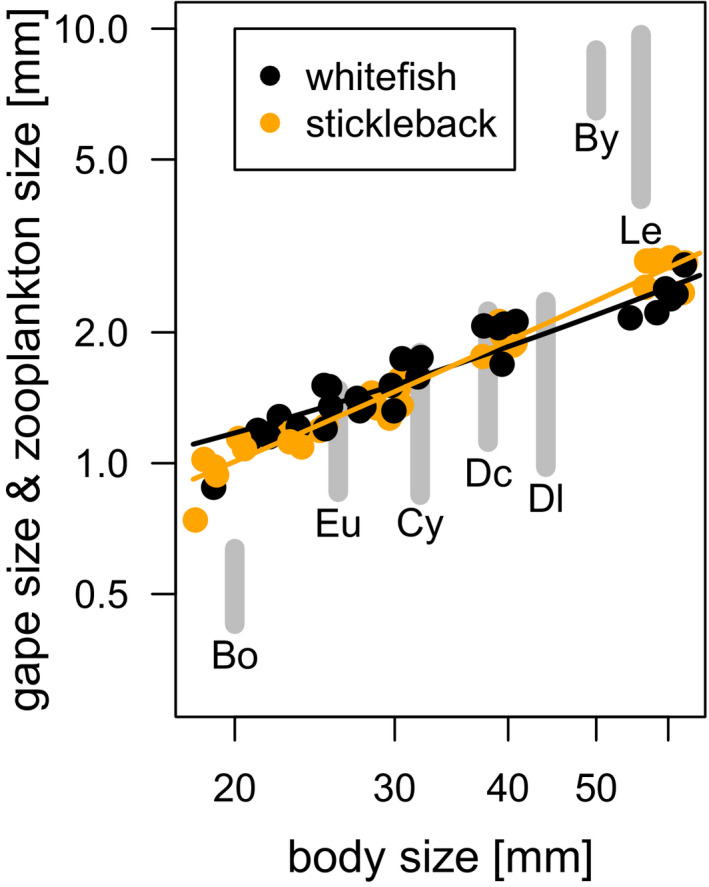
Relationships between gape size and body size for sticklebacks (orange) and whitefish (black). Lines show the predictions of ANCOVA with fish species as a covariate (*F*_1,56_ = 15.2, *p* < .001). Grey bars show the size range of the zooplankton species used in feeding experiments displayed with increasing maximum size along the *x*‐axis. Bo = *Bosmina* spp., Eu = *Eudiaptomus gracilis*, Cy = cyclopoid copepods, Dc = *Daphnia cucullata*, Dl = *Daphnia longispina*, By = *Bythotrephes longimanus* and Le = *Leptodora kindtii*. Gape size increased with fish length for sticklebacks according to: gape size [mm] = 0.10 (± 0.067) +0.045 (± 0.002) * fish length [mm], and for whitefish according to: gape size [mm] = 0.49 (± 0.08) +0.034 (± 0.002) * fish length [mm]

The gape size of both fish species increased from approximately 1 mm for 2 cm fish to ~2.5 mm for 6 cm fish (Figure 2). However, small sticklebacks had slightly smaller and large sticklebacks slightly larger gape sizes than similar size whitefish (ANCOVA, fish species x length interaction, *F*_1,56_ = 15.2, *p* < .001). Maximum prey dimensions of *Bythotrephes* and *Leptodora* exceeded the gape size also for the 6 cm fish, whereas all zooplankton species except for *Bosmina* exceeded the gape size of the 2 cm fish (Figure 2).

**FIGURE 3:**
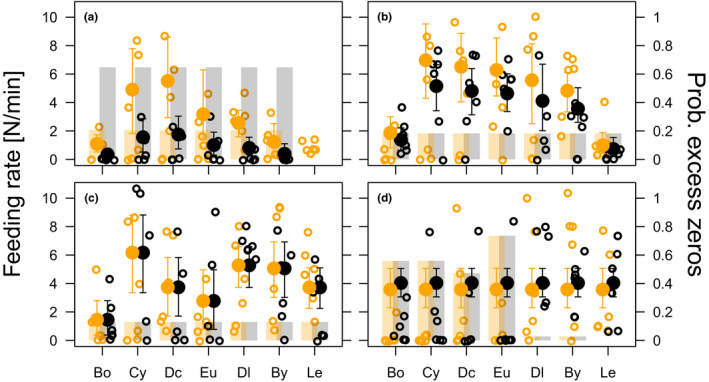
Observed feeding rates (open circles), predicted feeding rates (filled circles ± CI) and predicted percentages of non‐feeding fish (bars) of (a) 2 cm, (b) 3 cm, (c) 4 cm and (d) 6 cm sticklebacks (orange) and whitefish (black) on different zooplankton species. Predicted feeding rates and predicted percentage of excess non‐feeding fish are based on the ZINB models shown in Table 1. Bo = *Bosmina*, Cy = cyclopoid copepods, Dc = *Daphnia cucullata*, Eu = *Eudiaptomus*, Dl = *Daphnia longispina*, By = *Bythotrephes* and Le = *Leptodora*. Note that *Leptodora* was not provided to 2 cm whitefish, therefore it was excluded from model predictions

**FIGURE 4:**
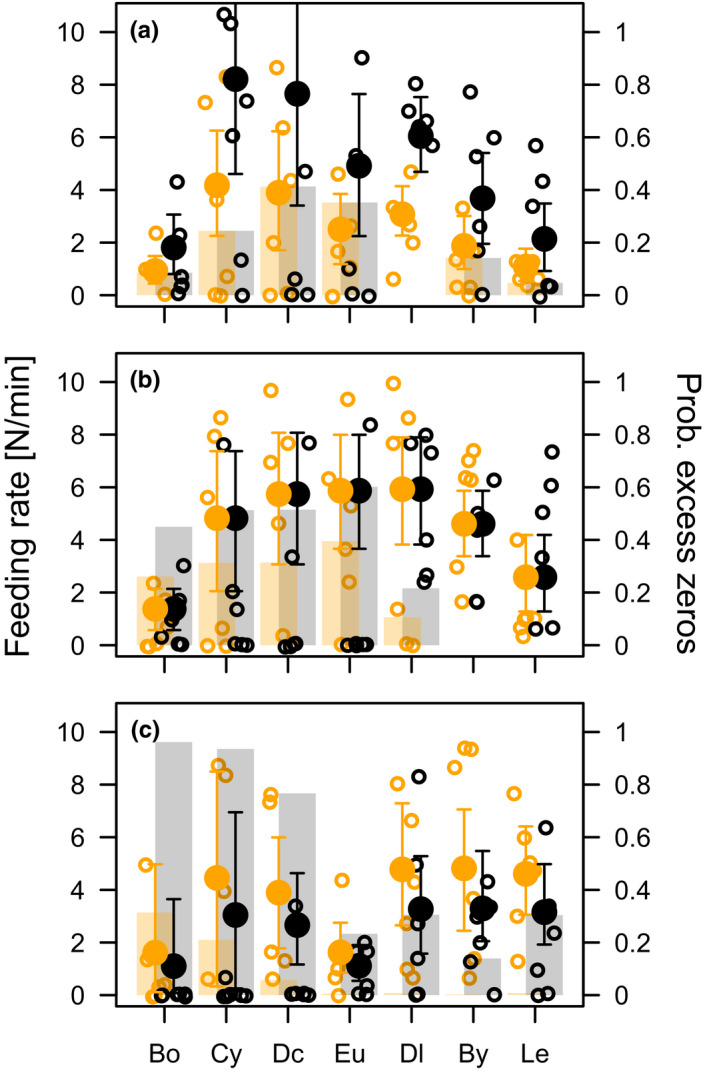
Observed feeding rates (open circles), predicted feeding rates (filled circles ± CI) and predicted percentages of non‐feeding fish (bars) of different sticklebacks (orange) and whitefish (black) size pairs on zooplankton. Predicted feeding rates and predicted percentage of excess non‐feeding fish are based on the ZINB models shown in Table 1. Fish size pairs correspond to the sizes of the two fish species typical for (a) July, 2 cm stickleback versus 4 cm whitefish, (b) August, 3 cm stickleback versus 6 cm whitefish, and (c) September, 4 cm stickleback versus 8 cm whitefish. Bo = *Bosmina*, Cy = cyclopoid copepods, Dc = *Daphnia cucullata*, Eu = *Eudiaptomus*, Dl = *Daphnia longispina*, By = *Bythotrephes* and Le = *Leptodora*

Feeding was observed in 251 (67.8%) of 370 feeding trials. Feeding rates differed in a complex way between prey species and partially also between the two fish species (Figures 3 and 4, Table 1). In the following, we distinguish between the feeding rates as predicted by the NB part of the ZINB model, and the probability of excess non‐feeding fish as predicted by the logit part of the ZINB model. With one exception (6 cm sticklebacks versus 6 cm whitefish), all fish size comparisons predicted prey‐specific feeding rates, i.e. prey ID contributed significantly to the negative binomial (NB) part of the ZINB models (Table 1). In addition, prey ID contributed four out of seven times to the logistic part of the ZINB models based on AIC. However, for the 2 cm stickleback versus 4 cm whitefish comparison, prey ID contributed to the best model but was not significant based on the likelihood ratio test (Table 1).

**TABLE 1:** List of best models predicting feeding rates of equal‐size and seasonal‐size whitefish and stickleback pairs (S, stickleback; W, whitefish)

Compared fish sizes (cm)	NB part of the model		Logistic part of the model	
	Fish ID	Prey ID	Fish ID	Prey ID
Same size comparisons				
2 cm S–2 cm W	χ^2^ = 13.4, df = 1***	χ^2^ = 18.9, df = 5**	χ^2^ = 9.97, df = 1**	
3 cm S–3 cm W	X	χ^2^ = 43.3, df = 6***		
4 cm S–4 cm W		χ^2^ = 14.7, df = 6*		
6 cm S–6 cm W	X			χ^2^ = 32.8, df = 6***
Same month comparisons				
2 cm S–4 cm W	χ^2^ = 12.3, df = 1***	χ^2^ = 29.0, df = 6***		x
3 cm S–6 cm W		χ^2^ = 20.4, df = 6**	x	χ^2^ = 21.6, df = 6**
4 cm S–8 cm W	X	χ^2^ = 13.1, df = 6*	χ^2^ = 24.3, df = 6***	χ^2^ = 21.6, df = 6**

Note

The *p*‐values for each predictor are indicated by asterisks (*p < .05, **p < .01, ***p < .001). The interaction between Fish ID and Prey ID never contributed to the best model. “x” indicates that the best model according to AIC included this predictor, but the likelihood ratio test suggest that the factor was not significant.

Fish species contributed five times to the NB part of the best models (Table 1), although significantly only for two comparisons (2 cm sticklebacks versus 2 cm whitefish, and 2 cm sticklebacks versus 4 cm whitefish). Fish ID also contributed three times to the logistic part of the best models, and two times significantly so based on likelihood ratio tests (2 cm sticklebacks versus 2 cm whitefish, and 4 cm sticklebacks versus 8 cm whitefish). For all comparisons, a ZINB model was preferred relative to a negative binomial model without a logit part.

Across all zooplankton species, except for *Leptodora* which was not used for 2 cm fish comparison (see *Methods*), 2 cm sticklebacks had approximately three‐fold higher feeding rates than 2 cm whitefish (Figure 3a). In addition, the percentage of excess non‐feeding fish was three‐fold higher for 2 cm whitefish (mean: 64.7%, CI: 39–82%) compared to 2 cm sticklebacks (mean: 20.5%, CI: 7–36%) (Figure 3a, Table 1).

Equally‐sized 3, 4 and 6 cm fish did not show significant differences in feeding rates between fish species (Table 1, Figure 3b–d), although 3 cm stickleback tended (*p* < .1) to have larger feeding rates compared to 3 cm whitefish (Figure 3b): 3 cm stickleback feeding rates were approximately one third larger compared to 3 cm whitefish feeding rates for the various zooplankton species. Equally sized fish larger than 2 cm did not differ in the percentage of excess non‐feeding fish. (Figure 3b–d).

Feeding rates of 4 cm whitefish were approximately twice the feeding rates of 2 cm sticklebacks (Figure 4a) with no differences in the percentage of non‐feeding fish (Table 1). The latter is in contrast to the model comparing 2 cm sticklebacks with 2 cm whitefish as many non‐feeding 2 cm whitefish but not 4 cm whitefish were observed. Consequently, the predicted feeding rates and probabilities of non‐feeding for 2 cm sticklebacks (Figure 3a vs 4a) differ whether these fish were compared in models with 2 cm whitefish or with 4 cm whitefish. Sticklebacks (3 cm) differed from 6 cm whitefish significantly neither in the feeding rates nor in the percentage of excess non‐feeding fish. Finally, for 8 cm whitefish percentage of excess zeros was larger compared to 4 cm sticklebacks, but feeding rates did not differ significantly between species (Table 1, Figure 4c).

Significant differences of feeding rates in respect to prey ID mostly resulted either from feeding on small zooplankton species or on large zooplankton species (Figures 3 and 4). For 2 cm fish, feeding rates on *D*. *cucullata* were approximately two‐fold and significantly higher than feeding rates of both fish on *Bosmina* (Figure 3a). Likewise, 3 cm fish had higher feeding rates on intermediate sized zooplankton compared to small *Bosmina* (exception *D*. *longispina*) and large *Leptodora* (Figure 3b). 4 cm fish had three to four‐fold lower feeding rates on *Bosmina* compared to cyclopoid copepods, *D*. *longispina* and *Bythotrephes* (Figure 3c). For 6 cm fish feeding rates did not differ between zooplankton species, however, more than 50% of fish did not feed when offered the small zooplankton (*Bosmina*, cyclopoid copepods, *D*. *cucullata* and *Eudiaptomus*), whereas almost all fish fed on the larger zooplankton (*D*. *longispina*, *Bythotrephes* and *Leptodora*) (Figure 3d).

Feeding rates of fish occurring in July (2 cm stickleback and 4 cm whitefish) were larger for cyclopoid copepods, *D*. *cucullata* and *D*. *longispina* compared to *Bosmina* and *Leptodora*. (Figure 4a). August fish (3 cm stickleback and 6 cm whitefish) feeding rates on *Bosmina* were lower as those *on D*. *cucullata*, *D*. *longispina*, *Eudiaptomus* and *Bythotrephes* (Figure 4b), whereas September fish (4 cm stickleback and 8 cm whitefish) feeding rates were higher for *Leptodora* compared to *Eudiaptomus* (Figure 4c).

## 4 DISCUSSION

Invasion or introduction of fish species may change both the species composition and the seasonal dynamics of the zooplankton community. Such changes may result for instance from differences in prey selectivity between native and invasive fish and/or changes in overall predation pressure after invasion (Beisner et al., 2003; Brooks & Dodson, 1965). In Lake Constance, the pelagic system became dominated by sticklebacks rather than the native whitefish during the 2010s (Eckmann & Engesser, 2019; Rösch et al., 2018). Based on morphology, whitefish should be a zooplankton specialist (Kottelat & Freyhof, 2007; Lazzaro, 1987) and stickleback a generalist (Kottelat & Freyhof, 2007; Morrow, 1980). In general, specialists feed at higher rates on selected prey species but consume a narrower range of prey types compared to feeding generalists (David et al., 2017; Layman & Allgeier, 2012). However, these predictions were not supported in our study; whitefish did not have higher feeding rates on various zooplankton species than sticklebacks. Despite their small size, 0+ sticklebacks were successful in feeding on large zooplankton such as the predatory cladocerans *Bythotrephes* and *Leptodora*. Furthermore, stickleback's feeding rate was similar to whitefish of the same size and comparable to feeding rates of the larger 0+ whitefish that would co‐occur in the lake with the smaller sticklebacks. We did not detect a large difference in feeding rates through ontogeny of either species except for lower feeding rates of the smallest 2 cm fish. Below, we discuss how these ex‐situ findings match with *in situ* lake factors like seasonality, fish hatching, and ontogenetic growth which affect the feeding abilities of fish.

Experimental conditions could have affected the results. Despite an acclimation period of a full day for fish in the experimental aquaria before introducing the zooplankton prey and allowing up to 5 min to adjust to a possibly slight disturbance associated with prey introduction, fish did not consume prey in one third of the feeding trials. Lack of feeding might have been caused by at least three different mechanisms: (1) fish might have been too small to feed on specific zooplankton species, i.e. gape limitation (DeVries et al., 1998), (2) fish may not react to, or chose to avoid, small prey perhaps due to insufficient energetic return (Sinervo, 1997), or (3) fish might not have been sufficiently acclimatized to the experimental setting (Melvin et al., 2017). The significance of the logistic parts of the zero‐inflated models shows that the percentage of fish not feeding was non‐randomly distributed across fish sizes, fish species and prey species. This suggests that non‐feeding was due to specific fish or prey traits rather than the experimental setup. Small fish may be expected to feed less, especially for the less developed and experienced whitefish (Braum, 1964; Lazzaro, 1987) see results for 2 cm fish). Likewise, larger fish (6–8 cm) might stop feeding on small zooplankton as low energetic gain from feeding on small prey items is likely (Osenberg & Mittelbach, 1989; Wanzenböck, 1995; Werner & Hall, 1974). This is supported by the significant effect of prey species for the larger fish: non‐feeding was mostly observed when given small prey species as expected based on optimal foraging theory. Nevertheless, we cannot exclude that larger whitefish were feeding at artificially low rates due to relatively small experimental aquaria as aquaria size may have limited their cruising behaviour.

Low feeding rates on small zooplankton was observed also in other studies. In a laboratory study on perch, maximum feeding rates on 0.5 mm zooplankton (*Daphnia* and *Bosmina*) was observed for ~5 cm perch, whereas maximum feeding on 1 mm zooplankton was observed for ~8 cm perch. Perch >13 cm consumed very few 0.5 mm zooplankton (Byström & García‐Berthou, 1999; Wahlström et al., 2000). In the field, adult whitefish’ stomachs rarely contain small zooplankters like copepods or *Bosmina* when larger zooplankton are present (Becker & Eckmann, 1992; Mookerji et al., 1998). Notwithstanding the reasons (ecological or methodological) for a large number of non‐feeding fish in our experiments, zero‐inflated models allowed us to analyse all feeding trials and therefore consider different mechanisms influencing feeding in our experimental setting. The suitability of zero‐inflated models in analysing feeding experiments of fish is especially evident when comparing model predictions with similar sized fish: For 2 cm fish, the major difference of observed feeding rates between fish species was the difference in the percentage of non‐feeding fish possibly reflecting developmental and experience differences between fish species (see above). For. 3 and 4 cm fish the percentage of non‐feeding fish was roughly 10% and there was neither a difference between fish species nor between zooplankton species. For 6 cm fish, there were also no fish species differences, but strong zooplankton species differences in the percentage of non‐feeding fish. These differences suggest that with increasing fish size the reason for non‐feeding has shifted from fish‐specific constraints towards zooplankton specific energy gains. Such insights would not have been possible if instead of using zero‐inflated models other approaches, e.g., excluding all non‐feeding fish or non‐parametric statistics would have been used.

Changes in prey‐specific feeding rates during ontogenetic growth of both fish species followed the seasonal succession of these species *in situ*. In Lake Constance small zooplankton species (copepods, *Bosmina*) usually dominate in early spring, whereas daphniids and the large cladoceran predators only appear in larger numbers from May/June onwards (Seebens et al., 2013; Straile, 2015). Hence 2 cm whitefish will not encounter the larger zooplankton tested. However, 4 cm whitefish fed successfully on the large zooplankton species. For sticklebacks, which spawn from May to July in Lake Constance (Gugele et al., 2020), larger zooplankton are already abundant when 0+ fish reach 2 cm and our feeding trials show that 2 cm sticklebacks can feed on large predatory zooplankton. The ability of both fish species to feed on *Bythotrephes* already at 2 or 3 cm length was surprising given the large spine of this prey, which is suggested to be an effective antipredator defence against small fish (Barnhisel & Harvey, 1995; Compton & Kerfoot, 2004; Miehls et al., 2014). The difference between our and previous studies might be a geographical one, as *Bythotrephes* in North America, where previous studies were performed (Barnhisel & Harvey, 1995; Compton & Kerfoot, 2004; Miehls et al., 2014) develops a larger spine compared to the middle European populations of *Bythotrephes* (up to 5.5‐times versus up to 3‐times larger than its body size, respectively) (Korovchinsky, 2015) and it is unclear if the North American species should be considered to be *B*. *longimanus* or the Scandinavian species *B*. *cederstroemi* (Korovchinsky & Arnott, 2019). In addition, at least 0+ European whitefish might be better adapted to feed on *Bythotrephes* compared to North American fish due to the difference in time of their co‐occurrence. *Bythotrephes* is native to Europe and was first described in 1860 based on a specimen from Lake Constance (Leydig, 1860), whereas it first appeared in Northern America in the early 1980s (Berg & Garton, 1994).

Although the length of predatory zooplankton exceeded the gape size of also the largest (6 cm) fish used for equal size comparison, the fish of this and smaller size could feed on them. The width of prey was not measured but was presumably small enough to pass through the fish mouth. However, we assume that when any of the dimensions of prey exceeds the gape size of fish, then fish’ handling time increases which leads to a decrease in feeding rate (Wanzenböck, 1995). Longer handling times were indeed observed for some fish feeding on *Leptodora* and *Bythotrephes*. However, feeding rates of fish (except the smallest fish sizes) on the large zooplankton were relatively high, which suggest that long handling time might be compensated by shorter search time.

Overall feeding rates of 0+ sticklebacks on zooplankton were surprisingly high relative to similar‐sized whitefish even though 0+ whitefish are considered specialised planktivores. These difference in feeding rates of small fish are unlikely explained by differences in gape size, as those differences were small relative to the range of zooplankton sizes tested. Unfortunately, there are no studies that directly compare whitefish and stickleback mouth morphologies related to prey capture kinematics and suction speeds. However, insights into the role of differences in functional morphology might be gained by comparing benthivorous versus planktivorous fish. Accordingly, the high feeding rate of sticklebacks on especially large zooplankton may result from its functional adaptations to benthic environments, i.e. high suction generation capacity (McGee et al., 2013), whereas planktivore morphological adaptations improve capabilities to capture small and evasive zooplankton (Lazzaro, 1987). The high stickleback feeding rates relative to whitefish might be also due to aquaria volumes, which might have restricted feeding of fish using a swim‐search strategy (whitefish) more than fish using a hover‐search strategy (sticklebacks). This might explain why only 4 cm whitefish were found to have higher feeding rates to 2 cm sticklebacks, whereas for 6 cm whitefish versus 3 cm sticklebacks no significant differences were found, and 8 cm whitefish consumed less prey than 4 cm sticklebacks. Also, sticklebacks as an invasive species might have acclimatized faster to the feeding trial environment compared to whitefish contributing to higher stickleback feeding rates in some size classes. An additional reason for the success of sticklebacks might be rapid evolutionary adaptations to planktivorous feeding. Although no study has investigated feeding differences between the littoral and limnetic sticklebacks in Lake Constance, adaptive radiation in other systems have shown that limnetic sticklebacks evolved a sustained and prolonged swimming performance (Law & Blake, 1996) as well as morphological adaptations to limnetic feeding (greater jaw protrusion, faster strikes) compared to littoral individuals, which allowed them to more successfully feed on small and evasive copepods, whereas littoral/benthic sticklebacks were superior in capturing larger prey (McGee et al., 2013).

Contrary to our expectations, experimental trials showed that 0+ sticklebacks can successfully feed also on large zooplankton already at 3 cm, making it unlikely that a switch from whitefish to stickleback dominance would result in large changes in the seasonality of predation on these larger prey. We also found that both 0+ sticklebacks and 0+ whitefish are able to feed successfully on the invertebrate predators, *Leptodora* and *Bythotrephes* even at 2 to 4 cm lengths, suggesting that increased relative importance of sticklebacks will not result in increased importance of invertebrate predation on the herbivorous zooplankton community. In contrast, the high feeding rates on large zooplankton suggest that 0+ sticklebacks will compete with larger whitefish for the preferred food of whitefish, i.e. large zooplankton (Becker & Eckmann, 1992) thereby contributing to the postulated negative effect of the stickleback invasion on the growth of 1+ to 4+ whitefish (Rösch et al., 2018).

## 5 CONCLUSIONS

We have shown that 0+ sticklebacks have similar or higher feeding rates than 0+ whitefish on various zooplankton groups even though sticklebacks are facultative, and whitefish obligate zooplanktivores. Hence, we did not find evidence for our 1^st^ hypothesis (similar sized whitefish have higher feeding rates) and only partial evidence for our 2^nd^ hypothesis (0+ whitefish have higher feeding rates than 0+ sticklebacks when co‐occurring as 0+ whitefish are larger than 0+ sticklebacks throughout the first year of life). Also, we did not find evidence for our 3rd hypothesis that co‐occurring fish sizes differ in their feeding rates on specific zooplankton species. Thus, sticklebacks´ feeding ability on zooplankton is not likely to limit their spread into the limnetic zone of the lake. The high feeding rate of even small sticklebacks on large zooplankton suggests that large zooplankton will experience additional predation pressure. Hence, stickleback invasion might contribute to a decline of the preferred food sources of native whitefish. Studying feeding interactions of juvenile invasive fish in important for estimating the impact of invasive fish species on native food webs.

## CONFLICTS OF INTEREST

The authors confirm no conflict of interest.

## AUTHOR CONTRIBUTIONS

**Žiga Ogorelec:** Conceptualization (equal); Formal analysis (equal); Investigation (lead); Writing – original draft (lead); Writing – review & editing (equal). **Lars G. Rudstam:** Supervision (supporting); Writing – review & editing (equal). **Dietmar Straile:** Conceptualization (equal); Formal analysis (equal); Funding acquisition (lead); Supervision (lead); Writing – original draft (equal); Writing – review & editing (lead).

## Data Availability

Data presented in this study are available within the KonDATA repository at https://doi.org/10.48606/8
